# Cannulation of the arteriovenous fistula in haemodialysis: a systematic review and narrative synthesis

**DOI:** 10.1007/s40620-025-02448-6

**Published:** 2025-11-05

**Authors:** Mayte Chocarro-Haro, Miren-Idoia Pardavila-Belio, Cristina Labiano, Andrea Navarrete, Jon Urretavizcaya, Raquel Sola-Freire, María Izal, Inés Díaz-Dorronsoro, Ana Choperena

**Affiliations:** 1 Clínica Universidad de Navarra, Pamplona, Spain; 2 Department of Community, Maternity and Pediatric Nursing, School of Nursing, University of Navarra, Campus Universitario, C/ Irunlarrea, 1, 31008 Pamplona, Spain; 3 IdiSNA, Navarra Institute for Health Research, Pamplona, Spain; 4 SEDEN, Spanish Society of Nephrological Nurses, Madrid, Spain; 5 Department of Nursing Care for Adult Patients, School of Nursing, University of Navarra, Campus Universitario, 31008 Pamplona, Spain; 6 Marjory Gordon Program, Boston, USA

**Keywords:** Haemodialysis, Vascular access, Arteriovenous fistula, Arteriovenous grafts, Cannulation

## Abstract

**Background:**

Arteriovenous fistulas (AVF) and arteriovenous grafts (AVG) are the preferred options for establishing vascular access in adult patients undergoing haemodialysis treatment. Although various official recommendations exist for AVF and AVG cannulation, a comprehensive, personalised approach to cannulation has yet to be proposed. This systematic review highlights existing knowledge gaps and identifies best practices by synthesising quality evidence on all components involved in AVF and AVG cannulation for haemodialysis.

**Methods:**

A search was conducted across the PubMed, CINAHL, Cochrane, Scopus and Web of Science databases for studies published between January 2016 and January 2023. This review followed the PRISMA statement and was registered with PROSPERO (CRD42024293288).

**Results:**

Twenty-four studies met the inclusion criteria and reported outcomes for 11,687 patients and 801 ward staff in 14 countries. Collectively, their results emphasized a person-centred approach, the importance of nurses’ and patients’ skills, and the need for continuous learning to enhance patient care. While recommendations varied, the implementation of the button-hole technique and innovative nurse-led devices such as plastic cannulas and point-of-care ultrasound guided cannulation were highly recommended.

**Conclusion:**

This systematic review highlights the importance of adopting a person-centred approach to managing patients undergoing haemodialysis. It also recommends the systematic assessment of vascular access and the continuous training for nurses and patients. Further research is needed to evaluate the cost-effectiveness of innovative, nurse-led tools in haemodialysis units.

**Graphical abstract:**

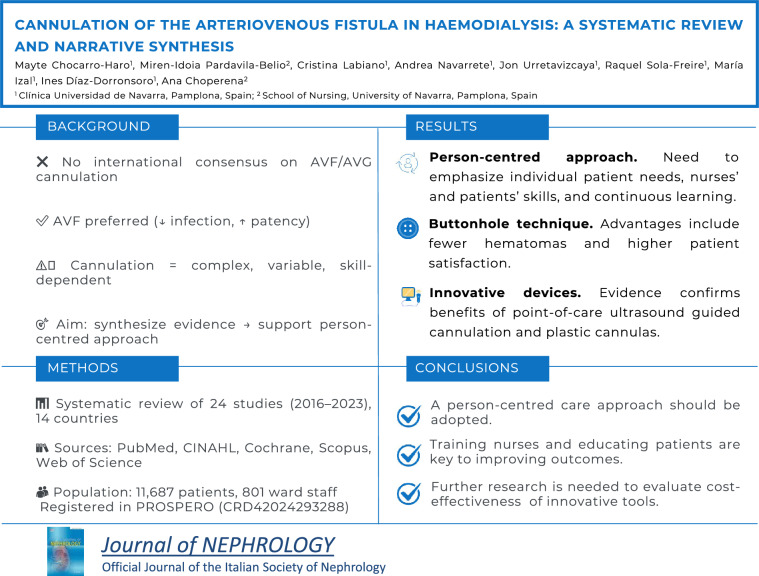

**Supplementary Information:**

The online version contains supplementary material available at 10.1007/s40620-025-02448-6.

## Introduction

Arteriovenous fistulas (AVFs) and grafts (AVGs) are the preferred vascular accesses for patients with chronic kidney disease undergoing haemodialysis (HD) (1). Evidence points to the superiority of AVF and AVG over catheters and to a lesser extent, AVF over AVG [1–3]. Evidence shows that AVF lasts longer, requires fewer interventions, has the lowest infection rates and manifests better patency than AVG or central venous catheter [2].

Nurses play a crucial role in cannulating AVFs and AVGs, significantly impacting the patients’ experience [4]. A thorough understanding of AVF and AVG performance could improve outcomes and inform decision-making [5]. However, patients and healthcare providers often encounter common cannulation-related complications associated with nursing cannulation skills [6, 7], haemostasis [3] and patient comorbidities [8].

AVF and AVG cannulation is a complex and multi-step process that can be executed in various ways. Numerous options are available for needle selection, choice of skin disinfectant, tourniquet use, needle insertion point, bevel positioning (i.e. up or down), and needle rotation and fixation [9]. Consequently, establishing reliable vascular access cannulation protocols that take into consideration substantial management differences between regions and countries is challenging [2]. Official recommendations exist for maintaining access patency, access type selection and preventing complications [10]. However, these vary in practice, given the individualised training necessary for AVF and AVG cannulation [11].

We examined the available evidence regarding all variations in AVF and AVG cannulation for HD, to provide a comprehensive systematic narrative regarding cannulation approach.

## Materials and methods

A systematic review with narrative synthesis was conducted following the guidelines established by Mays et al. [12] and Popay et al. [13]. The PRISMA statement guided the review [14, 15], and was registered with PROSPERO (CRD42024293288).

### Search strategy

The search was conducted from January 2016 to January 2023 across the PubMed, CINAHL, Cochrane, Scopus and Web of Science databases. In the PubMed database, we initially used specific headings to select terms indexed to each descriptor, followed by the Boolean expressions AND and OR to refine the search, which was then activated. An identical process was used for the CINAHL and EMBASE databases (Table 1). Additionally, we reviewed the citations and reference lists of the selected studies to ensure that no relevant papers were overlooked.

**Table 1:** Search strategy

Haemodialysis	**AND**	Fistula	**AND**	Cannulation
**OR**		**OR**		**OR**
Dialysis		Arteriovenous fistula		Catheterization
**OR**		**OR**		**OR**
Dialyses		Vascular fistula		Needling
**OR**		**OR**		
Haemodialyses		Vascular access		
**OR**		**OR**		
Haemodialyses		Arteriovenous graft		
**OR**				
Renal dialysis				
**OR**				
Renal dialyses				

### Eligibility criteria

We used qualitative, quantitative, mixed-method studies and systematic reviews to provide insight into stakeholder’s decisions when navigating the AVF and AVG cannulation process. Studies conducted in public and private hospitals/clinics with adult patients were included. We excluded all paediatric studies, studies of other, non-AVF or AVG cannulation methods, single-puncture cannulation studies, home dialysis studies, and studies that addressed specific aspects of fistula care without addressing cannulation, since these studies solely focused on pain assessment and/or treatment. Studies that addressed commercial purposes, those that strictly focused on cannulation complications or early cannulation issues, and those that focused on the COVID-19 pandemic and simulation studies were also excluded.

### Study selection

**Fig. 1:**
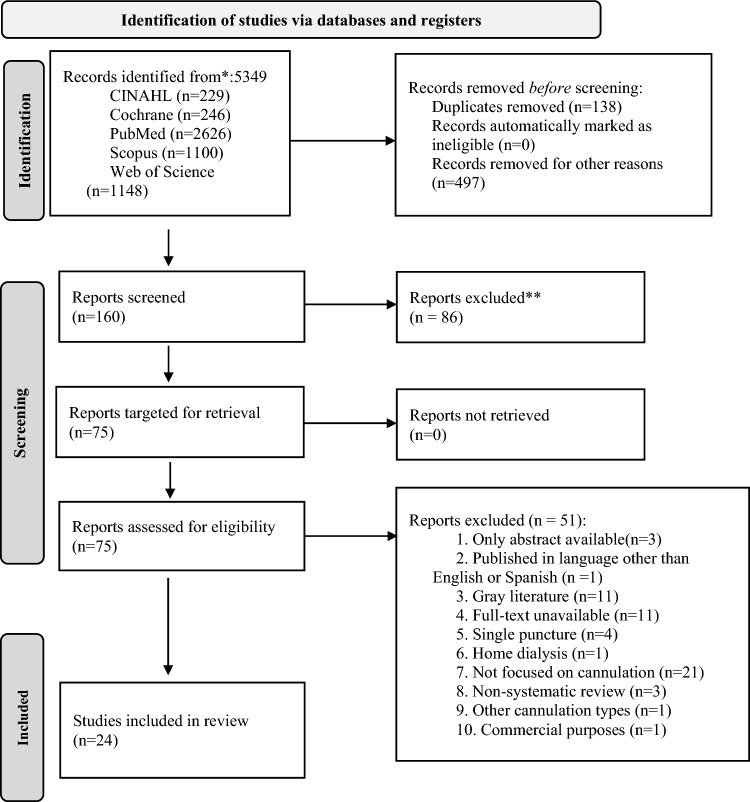
PRISMA2020 flow diagram for systematic reviews

All included articles in English and Spanish published from January 2016 to January 2023 were exported to Covidence software. After removing duplicates, two researchers (MCH and AC) independently assessed the titles and abstracts to determine their potential relevance for inclusion, and if there were discrepancies, a senior researcher (MPB) reviewed the articles. The eligible articles were then reviewed independently and blindly by three pairs of reviewers (MCH and MPB, MC and AN or MC and JU) to ensure a transparent selection process. If there were disagreements, a senior researcher (AC) resolved the conflicts. Following this selection, the full versions of the potentially eligible articles were extracted by MCH and reviewed by MPB and AC (Fig. 1).

### Quality appraisal

We used critical-appraisal-tools from the Joanna Briggs Institute (JBI) (https://jbi.global/critical-appraisal-tools) to determine the quality of each included study (Table 2).

**Table 2:** Study characteristics and quality

Main author, year, country	DOI	Design	Aim	N	Results	JBI
Bayoumi, 2020, Egypt	10.17532/jhsci.2020.887	Cross-sectional study	To identify the factors that lead to vascular access complications and develop holistic guidelines for cannulation techniques in Egypt	65 patients	Correlation between cannulation techniques and complications: *r* = 0.269 (*p* > 0.030)	Include
Choi, 2021a, Korea	10.1177/1129729820916579	RCT	To demonstrate the impact of the plastic cannula on maintaining stable blood flow and even reducing dynamic arterial and venous pressure despite a smaller diameter of inner introducer needle compared to metal needle in terms of arteriovenous graft integrity	16 patients	Brachial-artery blood-flow: PC 906.8 mL/min ± 3 52.6 mL/min; Metal needle 1313.3 mL/min ± 442.0 mL/min; *p* = 0.045	Include
Choi, 2021b, Korea	10.1159/000516212	RCT	To investigate whether implementing plastic cannulas for new AVFs affects cannulation failure rates and HD adequacy compared to traditional metal needles	96 patients	Time to achieve haemostasis at first needling (min): PC = 7.3 ± 1.3; MN = 8.8 ± 3.3; *p* = 0.015 Time from first needling to final success (days): PC = 6.3 ± 3.7; MN = 12.1 ± 4.8; *p* = 0.026 Initial cannulation failure rate: PC = 5 (11.1%); MN = 12 (26.7%); *p* = 0.051	Include
Coventry, 2019, Australia	10.1186/s12882-019-1373-3	Prospective cohort study	To identify patients, vascular access and nurse-related factors associated with unsuccessful VA cannulation	149 patients and 63 nurses	First cannulation success with 14G vs. 17G needles: OR = 7.65; 95%CI = 1.4 to 41.6; *p* = 0.02 First cannulation success with 15G vs. 17G needles: OR = 4.58; 95% CI = 1.12 to 18.8; *p* = 0.03 First cannulation success with no arterial needle rotation vs. yes: OR = 0.57; 95%CI = 0.36 to 0.91; *p* = 0.02	Include
Darbas- Barbé, 2016, Spain	10.4321/S2254-28842016000400008	Cross-sectional	The aim was to identify patients with a native arteriovenous fistula punctured by the area technique who were susceptible to changing to the staged technique based on the findings obtained from the Doppler ultrasound examination	63 patients	POCUS can be used to individualize and adjust the cannulation technique for each patient	Include
de Barbieri, 2021, Italy	10.1177/11297298211066763	Cross-sectional study	To investigate the use of plastic cannulas versus metal needles for cannulation in dialysis units and explore the implications of focusing on the side effects of cannulation	294 nurses	The use of metal needles was widespread all around the world (MN: 90–95% vs. PC: 5–10%) There was a greater proportion of adverse events in AVF during cannulation, haemodialysis treatment, needle removal and haemostasis in patients who were cannulated with MN	Include
Delgado-Ramírez, 2016, Spain	10.4321/S2254-28842016000300004	Systematic review	Factors influencing internal arteriovenous fistula survival and their association with the puncture technique	38 articles	There is no universal technique of choice; it depends on each patient There is a discrepancy in the advantages and disadvantages of the BH technique In some units BH is not common because of a lack of training among the nurses Some of the risks of BH are false tracks and bacterial growth	Include
Elias, 2018, France	10.1111/hdi.12539	Pilot study	To compare access recirculation rate in fistulas that were cannulated using two methodologies but with a constant distance of 2.5 cm	14 patients	Median recirculation rate when both needles upstream: 10% (range 6–13%) Median recirculation rate when both needles downstream: 9% (range 5–13%) Kt/*V* when both needles upstream: 1.29 (range 0.71–1.90) Kt/*V* when the arterial needle downstream: 1.31 (range: 0.68–1.85; *p* 5 0.20)	Include
Harwood, 2016, Canada	10.1093/ckj/sfv158	Qualitative study	To find attributes of excellence in nursing practice around AVF cannulation that could be used to cultivate successful interventions to promote changes to patient vascular access outcomes, thus creating a more positive environment/ culture for AVFs in the dialysis unit	18 patients	Patient-centred-care Teamwork Nurse self-awareness Opportunity and skill	Include
Marticorena, Canada, 2018	10.1177/1129729817747535	RCT	This study aimed to assess the feasibility of conducting an informative randomised controlled trial (RCT) comparing the two cannulation devices in the development of complications requiring diagnostic or surgical interventions	33 patients	Baseline number of events per patient: MN = 0.41 (0.7), PC = 1.25 (1.2), *p* = 0.019 Total complications: MN = 18 (1.06 ± 0.66), PC = 7 (0.44 ± 0.51), *p* = 0.005 Infiltration during HD with HD short/loss: MN = 14 (0.82 ± 0.64), PC = 5 (0.31 ± 0.48), *p* = 0.014 Number of patients with clinical complications: MN = 14 (83%), PC = 7 (44%), *p* = 0.021 Cost of cannulation device: MN = CAD$3932, PC = CAD$13,776 Cost of cannulation device CAD$3932 CAD$13,776 Total cost per study period: MN = CAD$58,932, PC = CAD$41,276 Estimated cost/patient-month: MN = CAD$6622, PC = CAD$3787	Include
Martins-Castro, Brasil, 2020	10.1590/2175-8239-JBN-2019-0031	Cross-sectional study	To evaluate the cannulation technique and to determine which factors are associated with each detail of the technique	260 patients	15G needles were more frequent than 16G needles (*p* = 0.015) When the distance between the needles was ≥ 5 cm, Kt/*V* was greater (When < 5 cm, Kt/*V* = 1.47 ± 0.28; When ≥ 5 cm, Kt/*V* = 1.61 ± 0.30; *p* < 0.001)	Include
Parisotto, 2017, Germany	10.5301/jva.5000617	Cross-sectional study	To investigate whether different aspects of arteriovenous fistula and graft cannulation have an effect on the development of acute access complications, which may affect VA survival	10,807 procedures	RL could be associated with a higher risk of multiple cannulation Risk of cannulation complications (CC) with 16G vs. 15G needles: OR = 1.305; 95% IC 1.016–1.676; *p* = 0.037 Risk of CC with 17G vs. 15G needles: OR = 4.245; 95% IC 2.548–7.072; *p* < 0.001 Risk of CC when rotating the needle: OR = 1.522; 95% IC 1.206–1.921; *p* < 0.001	Include
Özen, 2022a, Turkey	10.1111/hdi.13044	Single-blind crossover study	The aim is to investigate the effect of the orientation of the arterial needle bevel inserted into the AVF on puncture pain and post-removal bleeding time based on results averaged over multiple dialysis sessions	38 patients	Bleeding time = bevel down (minutes): 4.76 (± 0.98), bevel up (minutes): 5.89 ± 1.43, *p*-value < 0.001 Visual analogue scale: bevel up = 1.66 (1.66–2.33), bevel down = 1.16 (1–1.33), *p*-value < 0.001	Include
Özen, 2022b, Turkey	10.1111/jorc.12365	Cross-sectional	To determine the effect of various cannulation methods used for arteriovenous fistulas on dialysis adequacy	164 patients	Kt/*V* when antegrade needle: *B* = 0.107, OR = 0.164 *p* = 0.04, 95% CI 0.002–0.212	Include
Ren, 2016, China	ISSN:1940-5901/IJCEM0026135	Systematic review	To provide evidence for future BH use in China	10 studies	Aneurysm formation (events): BH = 4, RL = 20, *Z* = 3.36, *p* < 0.001 Thrombosis formation (events): BH = 10, RL = 21, *p* = 0.02 Stenosis formation (events): BH = 6, RL = 18, *Z* = 2.79, *p* = 0.005	Include
Sallée, 2021, France	10.1093/ckj/sfaa098	Cross-sectional study	To investigate the practices of cannulation and haemostasis and the knowledge of nurses and patients concerning maintaining a reliable AVF	150 supervisory nurses, 1538 nurses and 3588 patients	Method of controlling bleeding ≤ 10 min reported by nurses: dressing (88.7%) Method of controlling bleeding ≥ 10 min reported by nurses: dressing (84.3%) Dressings listed by nurses (bleeding ≤ 10 min): Coalgan/Coalgan H (47.7%) Dressings listed by nurses (bleeding ≥ 10 min): Coalgan/Coalgan H (65.3%)	Include
Schoch, 2020, Australia	10.1111/sdi.12909	Systematic review	To determine the circumstances in which renal nurses and technicians use POCUS, the barriers and facilitators; and evidence of the effects of POCUS in guiding assessment and cannulation	21 publications	Assessing new AVF maturation Identifying landmarks and abnormalities Assessing for alternate cannulation sites New AVF cannulation Difficult access cannulation Increasing cannulation accuracy	Include
Staaf, 2019, Sweden	10.1177/1129729818788811	Cohort study	To investigate whether the choice of cannulation technique–buttonhole with sharp or blunt needles–affected the development of arteriovenous fistula complications	49 patients	Local infection/AVF-year: sharp needle = 0.00; first sharp, then blunt = 0.14–0.03; mix sharp and blunt = 0.06	Include
Staff, 2023a, Sweden	10.1111/jorc.12448	Mixed-methods	To describe the preconditions for cannulation in arteriovenous fistulas	71 HD units	Planning cannulation: -Maturation and cannulation -Patient record -Education and experience Pre-cannulation: -Physical examination -Hygiene routines -Tourniquet -Choosing a cannulation site During cannulation: -How to needle -Type of needle -Angle - Fixating and adjusting Evaluating cannulation: -Blood-flow rate -Arterial and venous pressure Post cannulation: -Needle withdrawal -Haemostasis	Include
Staaf, 2023b, Sweden	10.1111/jocn.16454	Mixed-methods	To describe the basis for choosing a cannulation technique for arteriovenous fistula	29 units	Choice of cannulation technique Choice of good cannulation technique Preventive care and complications	Include
Wang, 2022, Taiwan	10.1097/MD.0000000000029597	Systematic review	The aim is to explore and compare the effects of BH puncture with RL puncture on vascular access, infection and pain by performing a systematic review and meta-analysis of studies with more rigorous design (RCT or CCT) or longer follow-up periods and to provide reliable research integration evidence as the basis for future clinical care	15 studies	Pain: BH = 165, RL = 178, OR/95% CI − 0.69 [− 1.78 to 0.4] Haematoma: BH = 281, RL = 299, OR [95% CI] 0.63 [0.4to 0.99] Thrombosis: BH = 211, RL = 201, OR/95%CI − 0.0.4 [0.2 to 0] Infection ≤ 6 months: BH = 10/170; RL = 5/198; OR [95% CI] 2.17 [0.76, 6.23] Infection > 6 months: BH = 29/378; RL = 8/367; OR [95% CI] 2.7 [0.92, 7.92]	Include
Wilson, 2018, Canada	10.1177/1129729818788811	Cohort study	To investigate whether the choice of cannulation technique–buttonhole with sharp or blunt needles–affected the development of arteriovenous fistula complications	252 nurses	When asked about important criteria for successful cannulation, 84.1% of the nurses agreed with ‘Patient level of comfort (i.e., pain with cannulation).’ Nurses agreed that the ‘Patient-centred approach during cannulation/ patient-centred care ‘friendliness’ of nurse’ is an important criterion for successful cannulation	Include

*AVF* arteriovenous fistula, *AVG* arteriovenous graft, *BH* buttonhole, *CAD $* Canadian dollar, *G* gauge, *HD* hemodialysis, *kt/V* clearance time volume, *MN* metal needle, *PC* plastic cannula, *POCUS* point of care ultrasound, *RCT* randomised controlled tiral, *RL* rope-ladder, *VA* vascular access

### Data abstraction and synthesis

We collected the data using Covidence and then analysed it to ensure it met our research objectives. We used a structured table to organise variables such as authors, year and country of publication, study aim, sample, study design, and relevant results (Table 2).

Following guidelines established by Mays et al. [12] and Popay et al. [13], we used a narrative synthesis approach to summarise the selected studies in a structured manner. We identified patterns and commonalities among the studies, which were thus organised into themes. Each paper was then evaluated in the context of each theme, each of which was further refined through an iterative process. Finally, we synthesised the evidence to provide a narrative relevant to the research question.

## Results

Twenty-four studies met the eligibility criteria. The key characteristics of these studies are outlined in Table 2. The 24 studies encompass data from 11,687 patients, 801 dialysis ward staff, 150 haemodialysis units, 29 local guidelines and 82 studies conducted across 14 countries. Overall, the quality scores of the studies were high, as indicated in Table 2. After assessing the methodological quality, one study with a low quality score was excluded.

AVF cannulation outcomes were categorised into five groups: (1) planning cannulation, (2) cannulation technique, (3) needle-related factors, (4) ultrasound-guided cannulation and (5) post-cannulation aspects.

### Planning cannulation

Wilson et al. [16]. analysed the opinions of both patients and professionals regarding AVF cannulation, and Harwood et al. [4] developed a qualitative study that explored factors contributing to cannulation success. Both studies highlighted the importance of person-centred care for successful cannulation.

In the former study, both patients and nurses agreed on the significance of the patient’s comfort level and the presence of direct support staff. In the latter study, nurses highlighted the importance of educating patients about the benefits of AVFs, and of the need for empathy. They also emphasised the significance of being sensitive to the patient’s emotional responses, approaching cannulation with a pre-planned strategy, and avoiding transferring their own nervousness to the patient. When nurses were asked about aspects of developing cannulation skills, a commonly expressed desire was to have more opportunities to practice the skill [4].

A mixed-method study by Staaf et al. [17] explored cannulation-related factors. They found that nurses perceived cannulation knowledge as being closely linked to experience. Nurses also found it important to keep their skills up to date by attending educational events, consulting with experts, and observing other expert nurses execute the procedure, and felt that teamwork and relying on colleagues were essential for successful cannulation [4].

Staaf et al. [17] analysed 29 local guidelines and recommended ‘the rule of 6’ (6 mm deep, 6 mm wide, blood flow of > 600 ml/min, 6 weeks to maturation) when describing factors indicating that a newly created AVF is ready for cannulation. The planning of AVF care and the management of AVF complications were referred to the *access nurse*, who is the key person responsible for informing patients. The local guidelines also emphasised the need to conduct a thorough physical examination of the fistula (inspection, auscultation, palpation) before cannulation. Staaf et al. [17] also highlighted the importance of hygiene routines for nurses and patients during the aseptic cannulation process (Table 3).

**Table 3:** Hygiene routines

	Nurse	Patient
Before treatment	apron, gloves, mask and/or visor during cannulation	Wash their arms
	- chlorhexidine 5 mg/ml or - 70% ethanol	
During cannulation	- disinfecting the whole AVF area from top to bottom or - local disinfection	
	- circular sweep or - disinfection in one straight direction	
	BH: scabs should be removed and the area disinfected before and after scab removal	
After treatment	- disinfect the cannulation site after needle withdrawal	

*AVF* arteriovenous fistula, *BH* buttonhole

### Cannulation technique

Staaf et al. [18] conducted a mixed-method study to elucidate the reasons behind the nurses’ choice of cannulation technique for AVF. The blunt needle buttonhole method was the most popular technique among nurses, while the least popular was area puncture. Nurses tended to prefer the blunt needle buttonhole method when the patient was also undergoing dialysis three times a week or more, if the cannulation area was short, and/or the patient feared needles. Conversely, the authors stated that the blunt needle buttonhole method should be avoided if the patient shows signs of poor hygiene or prefers a different technique.

Staaf et al. [17] and two systematic reviews with meta-analysis [19, 20] reported on the strengths of the buttonhole technique (e.g. low risk of infiltration, ease of placement, less pain, prolonged patency, fewer aneurysms, less stenosis, haematoma risk reduction and fewer thrombi). However, a systematic review conducted by Delgado-Ramírez et al. [21] found that the buttonhole technique is rarely implemented because knowledge about it is primarily theoretical and the nurses dislike it. Furthermore, the buttonhole technique is associated with some complications, such as the risk of false tracks and bacterial growth.

The rope-ladder technique has been described as a temporary solution if the buttonhole technique does not work for a particular patient [18]. However, a cross-sectional study conducted by Parisotto et al. [9] found a relationship between using the rope-ladder technique and an increased risk of multiple cannulations.

Wang et al. [20], Ren et al. [19] and Delgado-Ramírez et al. [21] found that infections were one of the most common cannulation-related complications. However, Wang et al. [20] and Ren et al. [19] found no statistically significant difference in the incidence of infections relative to puncture technique, while Delgado-Ramírez et al. [21] reported some discrepancies concerning the buttonhole technique.

A retrospective study conducted by Staaf and Uhlin [22] collected patient data and found that alternating between the blunt needle buttonhole technique and the sharp needle buttonhole technique significantly increased the incidence of local infections. Interdialytic bleeding was more common among patients cannulated with the sharp needle buttonhole technique compared to those cannulated with the blunt needle buttonhole technique.

Finally, Ren et al. [19] and Wang et al. [20] reported no statistically significant difference between using the buttonhole and rope-ladder techniques with regard to fistula survival.

### Needling-related factors

De Barbieri et al. [23] explored cannulation-related complications by interviewing nurses and found that metal needles were the most popular. Plastic cannulas were viewed as needing improvement, such as the need to incorporate wings (74.7%) and improve the blood visualisation system (39.8%). Pedreira-Robles et al. [24] conducted a cross-sectional study to analyse the nurses’ experiences with the use of plastic cannulas. They found that 42.3% of nurses reported that plastic cannulas were available at their workplace and 55.8% had received formal training on using plastic cannulas. However, a sizeable percentage of nurses mentioned cost when asked about the reasons for not using plastic cannulas (32.2%). A randomised controlled pilot study conducted by Marticorena et al. [25] assessed cannulation-related complications and found no significant differences between metal needles or plastic cannulas for blood pressure, pulse, blood-flow rate (Qa), dialysis circuit flow (Qb), urea reduction ratio (URR) and Kt/*V*. In a randomised open-label study, Choi et al. [26] found that patients cannulated with plastic cannulas showed lower dynamic pressures, allowing for a higher Qb. Choi et al. [27] also conducted a randomised controlled trial to determine whether implementing plastic cannulas for new AVFs affected cannulation failure rates and haemodialysis adequacy compared to traditional metal needles. They reported that initial cannulation failure was statistically significantly more frequent among patients cannulated with a metal needle. Lastly, Marticorena et al. [25] found more complications in the metal needles group. Furthermore, Choi et al. [27] found that plastic cannula use was associated with a shorter time to haemostasis at first needling. The local guidelines of haemodialysis units analysed by Staaf et al. [17] tended to recommend plastic cannulas when the AVF was new or if the patient was moving around, because of the reduced infiltration risk. De Barbieri et al. [23] also reported more adverse events in AVFs during cannulation, haemodialysis treatment, needle removal and haemostasis in patients cannulated with metal needles.

Regarding needle size, a cross-sectional study conducted by Martins-Castro et al. [28] analysed patient questionnaires to assess Kt/*V*, the distance between needles, the direction of the needles and needle size. Most respondents preferred 15G over 16G needles. Conversely, Sallée et al. [29] conducted a cross-sectional study that analysed questionnaires answered by nurses regarding haemostasis-related factors. The local guidelines reviewed by Staaf et al. [17] found 16G needles to be the most frequently used. Nevertheless, Staaf et al. found that most haemodialysis units recommended either 17G or 16G needles at the first cannulation. Parissotto et al. [9] analysed 10,807 cannulation procedures and found that 15G needles were most commonly used to cannulate AVFs and 16G needles were most widely used to cannulate AVGs. Most haemodialysis units included in Staaf et al.’s [18] mixed-methods study stated that 15G needles were saved for chronic (i.e. ‘non-acute’) AVFs. According to Parisotto et al. [9], cannulating with 16G needles or 17G needles was associated with a higher risk of complications, such as multiple cannulations, haemorrhage, haematoma, infiltration, etc. Along this line, a prospective cohort study by Coventry et al. [30] discussed cannulation episode success, cannulation-related complications and dialysis adequacy per session and found that cannulation was 265% more successful when a 14G venous needle was used instead of a 17G or a 15G, and 358% more successful than when a 17G needle was used.

Bayoumi and Khonji [31] conducted a cross-sectional study that analysed patient surveys. They found the most common needle placement was bevel up (72.4%). There was a consensus among the local guidelines reviewed by Staaf et al. [18], the cohort study carried out by Coventry et al. [30], the cross-sectional study of Sallée et al. [29], Parisotto et al. [9] and Staaf et al. [17]. However, in a single-blind crossover study, Özen et al. [32] found that patients experienced less pain when cannulated with the bevel facing down. Moreover, post-removal bleeding time was reduced when the bevel was pointed downwards. Parisotto et al. [9] found that rotating the needle post-puncture increased the risk of cannulation-related complications. This statement was also supported by Coventry et al. [30] who found that keeping the arterial needle upright, rather than rotating, increased the chances of cannulation success.

Elias et al. [33] prospectively explored cannulation-related factors. They found the median recirculation rate was 10% (range 6–13%) when patients were cannulated with anterograde needles compared to 9% (range 5–13%) when fistulas were cannulated with retrograde needles. Moreover, Özen et al. [35] found that Kt/*V* was lower when the needle was placed in the anterograde direction.

Dialysis units varied in their tourniquet recommendations [17]. When choosing a cannulation site, the researchers found that the arterial needle should be placed within 2 to 5 cm of the anastomosis. Some units recommended moving the next cannulation site 0.5–2 cm [17].

### Ultrasound-guided cannulation

The scoping review carried out by Schoch et al. [36] concluded that point-of-care ultrasound (POCUS) could be used to assess AVFs that have not reached the minimum required diameter of 6 mm to enable cannulation, and that POCUS was considered complementary to the nurses’ physical assessment. Furthermore, the use of ultrasound was associated with the potential to improve the cannulation experiences for patients with new AVFs, to increase the cannulation accuracy, and to increase dialysis pump speed tolerance. In fact, in a retrospective study by Darbas-Barbé et al. [34] that assessed the adjustment of the cannulation technique, the use of ultrasound findings allowed the switch from the area puncture technique to the rope-ladder technique.

### Post-cannulation

Needle placement can affect both venous and arterial pressure. In the study conducted by Staaf et al., the lower limit of arterial pressure during the first cannulation was set to − 100 mmHg, dropping to − 200 mmHg over time. During the first treatments, venous pressure was not raised above 100 mmHg, and once the AVF had matured, it was not to be raised above 150- or 200 mmHg [17].

Sallée et al. [29] found that the most common method for bleeding control was placing a dressing followed by a gloved finger. Staaf et al. [17] found that most units recommend removing both needles simultaneously at the same angle as their insertion, and applying no pressure before complete removal. All responding haemodialysis units recommended providing compression with sterile or clean gauze. Coalgan/Coalgan H (calcium alginate) was the most widely used dressing. In this study, the compression force applied to stop the bleeding for 10 or more minutes was reported as being strong in 77.2% of the cases, and when bleeding exceeded 10 min, the force was strong in 74.6% of cases. Staaf et al. recommended constant pressure, although the AVF thrill should be palpable the whole time. Finally, a growing trend toward using adjustable devices for applying pressure has been reported [51].

## Discussion

This is the first systematic review examining the available evidence regarding components involved in AVF and AVG cannulation for HD to provide a comprehensive narrative cannulation approach. By including diverse interventions, research designs, outcome measures and settings, this review provides a thorough understanding of current cannulation practices. Although similar studies have been conducted -a narrative review by Pinto et al. [2] that included clinical guidelines, a systematic review with narrative synthesis by Downey et al. [37] that addressed clinical signs monitoring, the Kidney Disease Outcomes Quality Initiative (KDOQI) that recommended general vascular access care [10] and the scoping review conducted by Harwood et al. [4] that focused on successful cannulation aspects- they did not address all the elements involved in the cannulation process in a narrative and integrated way.

**Fig. 2:**
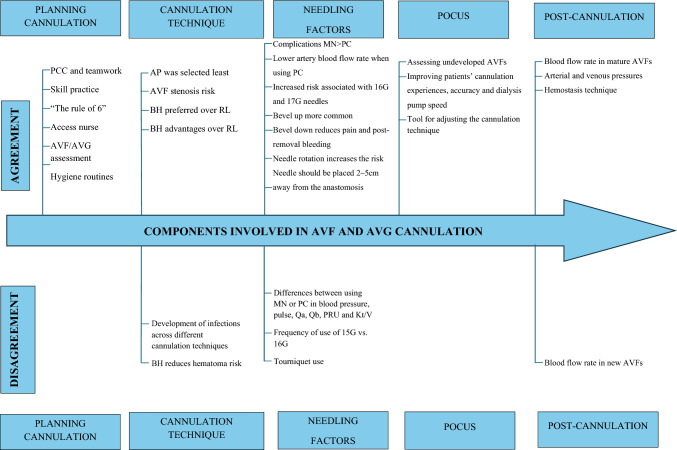
Components involved in AVF and AVG cannulation

Despite the significant challenges associated with cannulation for HD, little advice is given in the clinical units about how the procedure should be performed or regarding the training of nursing staff who cannulate. This has led to variations in cannulation [2]. The analysis of the studies involved in this systematic review identified notable differences between results when considering cannulation techniques and needling-related factors, offering less certainty as to the expected benefits and harms of different strategies; however, differences in pre-cannulation and post-cannulation aspects were less prominent (Fig. 2).

Considering the elements involved in the pre-cannulation process, there is a certain consistency in the results obtained in some of the studies included in this systematic review. On the one hand, these studies prioritised the person-centred care approach as an essential element for successful cannulation [4, 16]. Person-centred care has become a key global approach that seeks to address all factors of complex healthcare-related processes by helping nurses understand the patients’ personal life histories and health experiences [38]. Valentijn et al. [39] stated that person-centred care may reduce hospitalisation and improve blood pressure control compared with usual care. Lewis et al. [40] also identified practices related to person-centred care. They found that a complex interplay of prerequisites was necessary, including engaging patients and providers to jointly develop and evaluate a programme of ongoing care, and support clinical staff working to achieve better outcomes for their patients. The therapeutic person-centred care relationship gives patients a sense of feeling understood by nurses. Person-centred care can help restore hope, control the sense of helplessness, strengthen the positive aspects of health behaviours, improve medical care quality and help patients cope with challenges [41]. Furthermore, knowledge and desire for lifelong learning are commonly highlighted as important characteristics that nurses adopt to benefit their patients [4, 17]. Incorporating routines such as complete daily fistula examination and the ‘Rule of 6’ show promise for improving cannulation [17, 42, 43]. Patients examining their own fistulas on a daily basis has also been remarked upon [42].

Cannulation technique recommendations vary widely. Although conventional guidelines have traditionally presented the rope-ladder technique as the optimal and safest method [10, 52], this systematic review has predominantly favoured the buttonhole technique [17–20]. VanLoon et al. [44] also found that patients cannulated using the buttonhole technique experienced less haematoma formation, required less local anaesthetic cream and significantly fewer endovascular interventions. However, some risks have been identified, such as false tracks and bacterial growth [21]. Lyman et al. [45] also found that the buttonhole technique increased the risk of access-related bloodstream and local access-site infections. Nevertheless, as Collier et al. [46] stated, such risks can be mitigated through education programmess or strict aseptic guidelines. Given the advantages of the buttonhole technique over the other techniques, it is remarkable how infrequently it is implemented in dialysis units. Inadequate training of, and insecurity among, the nurses, as well as resistance by the patients are implementation barriers [21]. Ultimately, the technique should be custom-tailored to each patient, considering personal characteristics, arteriovenous access type and the haemodialysis team’s experience [47].

It is noteworthy that the area puncture technique was identified as the least popular option among nurses in the review by Staaf et al. [18]. Although this extended cannulation technique might be perceived as less complex in the short term as it does not require the skill of rotating sites or the careful establishment of a tunnel tract as in other methods, its long-term risks should be strongly acknowledged [47].

This systematic review raises many questions concerning needling-related factors. Concerning materials, the use of a plastic cannula may cause fewer complications than a metal needle [17, 23, 25, 27], possibly because of its smaller size which results in smaller puncturing holes and minimises haematoma formation [48]. Plastic cannulas have a higher substitution volume than metal needles [33]. Unfortunately, one of the most often reported obstacles to implementing plastic cannulas in haemodialysis units was the high cost compared to metal needles [25]. Long-term cost-effectiveness measures could be implemented and evaluated to achieve consensus regarding other needling-related factors, such as needle size [7–9, 9–18, 28, 30], placement [9, 18, 30, 31], direction [33, 35] and compression use [17]. Again, person-centred care requires an individualised approach to haemodialysis vascular access that considers each patient’s unique balance of risks and benefits [6].

Several studies in our systematic review recommended ultrasound-guided cannulation (POCUS) [34, 36]. However, these devices remain uncommon in haemodialysis units and require nurses to have specialised training. Some facilities use POCUS only in cases of puncture-related complications [42], as its high cost is seen as a barrier to general implementation [49]. In any case, more research is needed to position POCUS as a valuable tool for improving fistula cannulation [50].

Post-cannulation studies have highlighted the importance of appropriate needle removal [17, 29]. Traditionally, it was assumed that the most effective method for applying pressure to achieve haemostasis post-needle removal was direct, two-finger pressure applied by the patient. However, there has recently been a growing trend toward using adjustable devices for applying pressure [51]. Ultimately, studies show a consensus in favour of developing post-cannulation individualised care.

Without general reliable AVF and AVG guidelines, cannulation and post-cannulation procedures can be challenging. From a person-centred care approach, these procedures require continuous training, patient education and updating of nursing skills. Nurses should assess, plan, implement and evaluate the care given to patients before, during and after cannulation to provide individualised care, maintain cannulation competency and deal with complications properly. Patients should also be instructed on vascular access care, receive structured training under the nurses’ supervision and perform daily physical AVF examinations with nurses.

Awareness of costs and outcomes is essential for making value-based healthcare decisions and preventing complications. The implementation of innovative, nurse-managed devices for cannulation such as plastic cannulas and POCUS has the potential to enhance confidence when undertaking more challenging techniques. It is imperative that further research be conducted to evaluate the cost-effectiveness and cost–benefit of these innovative tools in haemodialysis units. This study has some limitations. Only studies published in English and Spanish were included for review. Furthermore, data not published in indexed scientific journals, such as local guidelines, were omitted. This synthesis used generalised results to describe various interventions and designs; therefore, our findings may not apply to every patient or clinical scenario. Finally, we only included challenges analysed in the selected articles. Other barriers may emerge over time.

## Conclusions

This review synthesises available evidence regarding  currently reported AVF and AVG cannulation variables. Although pre- and post-cannulation differences are less prominent, there is a notable discrepancy between the cannulation technique and the needle-related factor results. Instead of developing generic protocols or guidelines, there is an urgent and unmet need for personalised, case-oriented frameworks that take a person-centred care approach to managing patients undergoing haemodialysis. Our results reaffirm the importance of systematic assessment of the AVF and continuous training of nurses and patients. Evaluation of the cost-effectiveness of innovative nurse-managed devices such as plastic cannulas and POCUS is highly recommended.

## Supplementary Information

Below is the link to the electronic supplementary material.

Supplementary file1 (DOCX 269 KB)

## Data Availability

This is a narrative review article and does not include any original data. All data discussed are derived from previously published sources, which are appropriately cited in the reference list. No new data were generated or analysed during this study.
